# HIV-1 drug resistance and associated risk factors in patients with antiretroviral therapy failure in Chongqing, China, 2019–2023

**DOI:** 10.1371/journal.pone.0342301

**Published:** 2026-02-24

**Authors:** Huizheng Zhang, Ping Wu, Wei Ye

**Affiliations:** 1 Department of Clinical Laboratory, Chongqing Public Health Medical Center, Chongqing, China; 2 Department of Tuberculosis, Chongqing Public Health Medical Center, Chongqing, China; 3 Department of Physical Medicine and Rehabilitation, Chongqing General Hospital, Chongqing University, Chongqing, China; NIH: National Institutes of Health, UNITED STATES OF AMERICA

## Abstract

**Background:**

The emergence of drug resistance poses a major challenge to the long-term efficacy of antiretroviral therapy (ART) in managing HIV-1 infection. A comprehensive understanding of the prevalence and determinants of HIV-1 drug resistance mutations (DRMs) among patients with treatment failure in Chongqing, China, remains limited.

**Objectives:**

This study sought to characterize the prevalence of HIV-1 DRMs and to identify factors associated with drug resistance in patients experiencing ART failure in Chongqing between 2019 and 2023.

**Methods:**

We conducted a retrospective analysis of individuals living with HIV/AIDS who had received ART for at least six months and exhibited virologic failure(VF) (viral load(VL) > 1000 copies/mL). Genotypic resistance testing was performed on plasma samples. Associations between potential risk factors and the presence of DRMs were evaluated using univariate and multivariate logistic regression models.

**Results:**

Of the 1,522 patients with ART failure included in the study, amplifiable partial pol and integrase (INT) gene sequences were obtained from 1,426 and 653 specimens, respectively. DRMs were identified in 66.55% (949/1,426) of the patients. The predominant HIV-1 subtype was CRF07_BC (50.14%). Class-specific DRM prevalence was highest for non-nucleoside reverse transcriptase inhibitors (NNRTIs) at 61.15%, followed by nucleoside reverse transcriptase inhibitors (NRTIs) at 44.60%, integrase strand transfer inhibitors (INSTIs) at 6.13%, and protease inhibitors (PIs) at 5.39%. The most commonly observed mutations were M184V/I (38.00%) for NRTIs, K103N/S/H (24.96%) for NNRTIs, and M46I (1.54%) for PIs. Multivariate analysis confirmed that lower baseline CD4 + T-cell counts and initiation of treatment with an NNRTI-based regimen were independently associated with the development of DRMs.

**Conclusions:**

Our study reveals a high prevalence of HIV-1 drug resistance among patients with treatment failure in Chongqing, with low baseline CD4 + T-cell counts and NNRTI-based initial regimens identified as key risk factors. These findings underscore the urgency of optimizing first-line therapy by prioritizing dolutegravir (DTG)-based regimens, with protease inhibitor (PI)-based regimens as a practical alternative. Furthermore, we recommend implementing timely genotypic resistance testing, ideally within 4 weeks upon confirmed virologic failure, to guide effective regimen switching and curb the spread of drug resistance.

## Introduction

The HIV/AIDS epidemic continues to pose a significant public health challenge, particularly in low- and middle-income countries [1]. Currently, ART remains the most effective strategy to prevent new HIV infections and decrease the risk of AIDS-related deaths. By the end of 2024, more than 77% of the estimated 39 million people living with HIV globally were receiving ART [1]. Although ART has led to a reduction in mortality rates, the viral suppression rate among those receiving ART remains at 73% [1]. The “95-95-95” targets proposed by UNAIDS to end the AIDS epidemic have not yet been widely achieved.

In China, the National Free Antiretroviral Treatment Program (NFATP), launched in 2003, aligns with the national AIDS control policy of “Four Frees and One Care,” facilitating the expansion of ART access [2]. During the 2019−2023 study period, China’s ART strategy underwent a significant transition from traditional NNRTI-based first-line regimens (e.g., TDF + 3TC + EFV) toward integrase strand transfer inhibitor (INSTI)-based core regimens, aligning with the country’s continued progress toward the UNAIDS “95-95-95” targets [3,4]. Initially, dolutegravir (DTG) was primarily used as a second-line agent for salvage therapy following virological failure or for optimized treatment after clinical evaluation. With increasing availability and evidence, DTG has become the preferred INSTI and been integrated into guidelines for a broader range of scenarios, including first-line treatment, regimen switching, special populations, and simplified therapy. By the end of 2023, over 1.28 million patients had received ART across the country [5]. Although there has been an expansion in treatment coverage and a consistent reduction in AIDS-related mortality, HIV remains the primary cause of death among infectious diseases in China, and AIDS-related deaths continue to present a significant public health challenge.

Antiretroviral drug resistance mutations are an important cause of VF [6], which not only reduces the efficacy of ART [7,8] but also increases the risk of opportunistic infections and the emergence of transmitted resistance [9–11]. Understanding the prevalence of drug resistance among patients with HIV experiencing ART failure, characterizing the spread of HIV-1-resistant strains, and identifying associated risk factors, such as sociodemographic factors (e.g., gender, marital status, level of education, etc.) and clinical factors (e.g., treatment adherence, WHO staging, drug toxicity, ART regimen, CD4 + T-cell counts) can help define the resistance profile. The timely identification of predictors for treatment efficacy can improve drug utilization, minimize unwanted side effects of ART, and prevent the emergence of drug-resistant strains. Additionally, given the high cost of ART drugs, recognizing risk factors can reduce the financial burden and assist clinicians and public health officials in formulating strategies to mitigate treatment failure.

Chongqing, a major metropolitan region in southwestern China, plays a pivotal role in the national HIV prevention and control efforts. By the end of 2023, approximately 68,000 people were living with HIV in Chongqing, with 24,000 cumulative AIDS-related deaths reported [12]. Nevertheless, a comprehensive and systematic analysis of HIV drug resistance patterns and risk factors associated with ART failure is still lacking for this region. Furthermore, studies investigating VF across the three major antiretroviral drug classes, particularly in the context of increasing use of integrase strand transfer inhibitors (INSTIs), remain scarce. Consequently, a detailed regimen-specific resistance profile, especially for INSTIs, has not been established for this population. This study aims to address this critical knowledge gap by characterizing the spectrum of drug resistance mutations (DRMs) and identifying associated risk factors among patients experiencing ART failure in Chongqing.

Understanding the current status of drug resistance and the risk factors for ART failure among HIV-1-infected individuals in Chongqing is crucial for the timely assessment of treatment efficacy. Such knowledge is essential to ensure the long-term success of ART, facilitate effective monitoring, enable early detection of treatment problems, support the development of targeted interventions, and ultimately improve the management of treatment failure.

## Methods

### Study design and population

This retrospective observational cohort study was conducted among HIV-infected patients receiving ART at the Chongqing Public Health Medical Center, China. VF was defined as a viral load (VL) ≥1000 copies/mL after at least 6 months of ART initiation.. Data were collected from patients who visited the center between January 2019 and December 2023, who met the criteria for VF, and had available HIV drug resistance outcomes. In China, HIV drug resistance testing is recommended for patients with confirmed virological failure to guide regimen switching, as per the national guidelines [13].

### Ethical approval and data handling

The study protocol was approved by the Ethics Committee of Chongqing Public Health Medical Center (Approval No. 2024-046-01-KY). The research data were accessed for analysis on October 11, 2024.

During the initial data collection from the hospital’s case management system, the research team had access to information that could identify individual participants. Immediately after data compilation and before any statistical analysis, all personally identifiable information was permanently removed and replaced with a unique, anonymized study code. Therefore, during the data analysis phase, the authors had no access to any information that could identify individual participants. Given the retrospective nature of the study and the use of fully anonymized data for analysis, the ethics committee waived the requirement for written informed consent.

### Information collection

Patient information was obtained from the case management system, including demographic information (gender, age), ART-related information (initial ART regimen, ART duration), and virologic (HIV-1 RNA) and immunologic (baseline CD4 + T-cell counts) indicators.

### HIV-1 RNA extraction, amplification, and sequence analysis

Testing was performed as part of routine diagnostics in a single, centralized laboratory. The laboratory follows China CDC national guidelines and has consistently received “Excellent” ratings in national proficiency testing, ensuring quality assurance.Plasma was centrifuged at 3000 × g for 15 min. HIV-1 RNA was extracted using a viral nucleic acid extraction kit (Jiangsu Shuoshi Biotechnology Co., Ltd.), with positive and negative controls included. RNA was stored at −80°C with ≤3 freeze–thaw cycles. Nested RT-PCR was performed to amplify the protease, reverse transcriptase, and integrase regions of the HIV-1 pol gene. First-round RT-PCR used the HiScript® II One Step RT-PCR Kit with gene-specific primers. Second-round PCR used the Ace Taq Kit with nested primers. Amplified products were verified by agarose gel electrophoresis and sent for Sanger sequencing.

### Subtyping and phylogenetic analysis

Sequences were assembled and edited using ChromasPro and BioEdit. HIV-1 genotyping was performed using the NCBI Viral Genotyping Tool and REGA HIV-1 Subtyping Tool v3.0, with HXB2 (K03455) as reference. Phylogenetic analysis was conducted in MEGA 11.0 using the Neighbor-Joining method with 1000 bootstrap replicates.

### Drug resistance interpretation

HIV-1 pol sequences were analyzed using the Stanford HIV Drug Resistance Database (HIVdb Program, version 9.0) [14]. Resistance levels were categorized as: Susceptible (S, score 0–9), Potential Low-level (P, 10–14), Low-level (L, 15–29), Intermediate (I, 30–59), and High-level (H, ≥ 60). Low-level resistance and above (L, I, H) were defined as clinically resistant.

### Laboratory protocol deposit

The detailed laboratory protocol for HIV-1 RNA extraction, amplification, and sequencing has been deposited in protocols.io and is publicly available under DOI: [dx.doi.org/10.17504/protocols.io.rm7vz9495gx1/v1](https://dx.doi.org/10.17504/protocols.io.rm7vz9495gx1/v1).

### Statistical analysis

Statistical analysis was carried out using SAS 9.4 (version 9.4 for Windows, SAS Institute, Inc., Cary, NC, USA). Data with a skewed distribution were presented as median (interquartile range [IQR]), and the Wilcoxon rank-sum test was used for group comparisons. Categorical variables were presented as counts or percentages. The Chi-squared test and Fisher’s exact test were applied for comparisons between groups, while univariate logistic regression was used to analyze risk factors. All statistical tests were two-tailed, with P < 0.05 considered statistically significant.

## Results

### Clinical data analysis

A total of 1,522 people living with HIV/AIDS (PLWHA) who experienced VF were included in the final analysis. The median age was 49.00 years (range,0.58–90.00). The cohort was predominantly male (76.35%; 1,162/1,522) and of Han ethnicity (72.01%; 1,096/1,522). In terms of marital status, 32.33% (492/1,522) were married, while 28.58% (435/1,522) were either unmarried, divorced, or widowed. Non-workers made up 48.95% (745/1,522) of the cohort, followed by farmers (12.35%, 188/1,522), workers (9.40%, 143/1,522), and students (0.79%, 12/1,522). The documented transmission routes were as follows: heterosexual contact (29.43%; 448/1,522), men who have sex with men (MSM, 7.42%; 113/1,522), people who inject drugs (PWID, 1.31%; 20/1,522), mother-to-child transmission (0.66%; 10/1,522), and unknown (61.17%; 931/1,522). Immunologically, the median baseline CD4 + T-cell count was 81.00 cells/μL, and the median baseline HIV-1 VL was 95,284 copies/mL (Table 1).

**Table 1:** Baseline characteristics of people living with HIV experiencing virologic failure in Chongqing, China, 2019–2023.

Characteristics	Total (*n* = 1522)	2019 (*n* = 153)	2020 (*n* = 295)	2021 (*n* = 398)	2022 (*n* = 298)	2023 (*n* = 378)
**Sex**						
**Male**	1162(76.35)	122(79.74)	230(77.97)	308(77.39)	225(75.50)	277(73.28)
**Female**	360(23.65)	31(20.26)	65(22.03)	90(22.61)	73(24.50)	101(26.72)
**Age at diagnosis, years, median, (IQR)**	49.00(0.58-90.00)	45.00(1.00-85.00)	44.00(0.58-80.00)	50.00(6.00-86.00)	50.00(5.00-82.00)	52.00(7.00-90.00)
**Ethnicity**						
**Han**	1096(72.01)	118(77.12)	185(62.71)	251(63.07)	220(73.83)	322(85.19)
**Other**	44(2.89)	7(4.58)	8(2.71)	14(3.52)	9(3.02)	6(1.59)
**Unknown**	382(25.10)	28(18.30)	102(34.58)	133(33.42)	69(23.15)	50(13.23)
**Education**						
**Illiterate or Primary**	286(18.79)	29(18.95)	45(15.25)	91(22.86)	75(25.17)	46(12.17)
**Middle school**	252(16.56)	34(22.22)	56(18.98)	69(17.34)	57(19.13)	36(9.52)
**College degree or above**	88(5.78)	16(10.46)	25(8.47)	20(5.03)	21(7.05)	6(1.59)
**Unknown**	896(58.87)	74(48.37)	169(57.29)	218(54.77)	145(48.66)	290(76.72)
**Occupation**						
**Students**	12(0.79)	0(0.00)	1(0.34)	2(0.50)	3(1.01)	6(1.59)
**Farmers**	188(12.35)	14(9.15)	29(9.83)	38(9.55)	44(14.77)	63(16.67)
**Workers**	143(9.40)	27(17.65)	29(9.83)	32(8.04)	26(8.72)	29(7.67)
**Non-workers**	745(48.95)	81(52.94)	124(42.03)	174(43.72)	147(49.33)	219(57.94)
**Unknown**	434(28.52)	31(20.26)	112(37.97)	152(38.19)	78(26.17)	61(16.14)
**Marital status**						
**Married**	492(32.33)	63(41.18)	88(29.83)	119(29.90)	111(37.25)	111(29.37)
**Unmarried**	213(13.99)	29(18.95)	46(15.59)	46(11.56)	40(13.42)	52(13.76)
**Divorced or widowed**	222(14.59)	25(16.34)	36(12.20)	63(15.83)	52(17.45)	46(12.17)
**Unknown**	595(39.09)	36(23.53)	125(42.37)	170(42.71)	95(31.88)	169(44.71)
**Transmission category**						
**MTCT**	10(0.66)	0(0.00)	3(1.02)	0(0.00)	3(1.01)	4(1.06)
**IDU**	20(1.31)	6(3.92)	4(1.36)	4(1.01)	2(0.67)	4(1.06)
**HSX**	448(29.43)	53(34.64)	71(24.07)	128(32.16)	102(34.23)	94(24.87)
**MSM**	113(7.42)	14(9.15)	29(9.83)	26(6.53)	21(7.05)	23(6.08)
**Unknown**	931(61.17)	80(52.29)	188(63.73)	240(60.30)	170(57.05)	253(66.93)
**Baseline CD4** ^+^ **T-cell count(cells/μL)** **median** **(min/max)**	81.00(1.00-4224.00)	49.00(1.00-804.00)	69.50(1.00-1184.00)	84.00(1.00-579.00)	82.00(4.00-2578.00)	105.00(1.00-4224.00)
**Baseline HIV VL** **(copies/mL plasma), median (min/max)**	95284.00(20.00-8520000000.00)	149337.00(31.70-18000000.00)	90127.00(20.00-34500000.00)	79800.00(20.00-273000000.00)	139500.00(378.00-8520000000.00)	74200.00(20.00-85200000.00)

IQR, Interquartile Range; MTCT, mother to child transmission, HSX, heterosexual orientation, MSM, men who have sex with men, IDU,Intravenous drug use

### Prevalence of HIV drug resistance mutation

Of the 1,522 patients who experienced ART failure, genotypic resistance testing successfully generated partial pol gene sequences for 1,426 individuals and integrase (INT) gene sequences for 653 individuals. The INT sequence data constituted a subset of the pol sequence dataset. HIV-1 subtyping, validated by phylogenetic analysis (S1 Fig), showed that CRF07_BC was the predominant subtype (50.14%, 715/1,426), followed by CRF01_AE (24.96%, 356/1,426), CRF08_BC (12.76%, 182/1,426), B (2.03%, 29/1,426), and CRF55_01B (1.82%, 26/1,426). (Table 2)

**Table 2:** Prevalence of drug resistance mutations across HIV-1 subtypes among patients with virologic failure in Chongqing, 2019–2023.

variable	Patients, *n* (%)	DRMs, *n* (%)	OR (95% Cl)	*P*
**B**	29(2.03)	21(72.41)	1.750(0.764 ~ 4.009)	0.186
**CRF01_AE**	356(24.96)	242(67.98)	1.415(1.079 ~ 1.856)	**0.012**
**CRF07_BC**	715(50.14)	438(61.26)	1.00(ref)	
**CRF08_BC**	182(12.76)	127(69.78)	1.456(1.004 ~ 2.112)	**0.048**
**CRF55_01B**	26(1.82)	25(96.15)	16.623(2.244 ~ 123.12)	**0.006**
**Others**	118(8.27)	96(81.36)	2.650(1.805 ~ 3.889)	**<0.001**
**Total**	1426(100)	949(66.55)		

Univariate logistic regression analysis was performed. OR, Odds Ratio; CRF, Circulating recombinant forms; DRMs, Drug resistance mutations; HIV-1, Human deficiency virus type 1; PLWH, People living with HIV.“Others” include recombinants including CRF52_01B, C, CRF68_01B, CRF78_cpx, CRF59_01B, CRF85_BC, A, and CRF62_BC.

Drug resistance mutations (DRMs) were identified in 66.55% (949/1,426) of the patients. The DRM prevalence varied significantly among subtypes (P < 0.05), with CRF55_01B exhibiting the highest rate (96.15%, 25/26) and CRF07_BC the lowest among the major subtypes (61.26%, 438/715) (Table 2).

Resistance to NNRTIs was the most frequent (61.15%, 872/1,426), followed by resistance to NRTIs (44.60%, 636/1,426). In contrast, resistance to PIs (5.39%, 77/1,426) and INSTIs (6.13%, 40/653) was less common. The most prevalent DRM was M184V/I (38.00%) for NRTIs, while K103N/S/H (24.96%) was the most common for NNRTIs. For PIs and INSTIs, the predominant mutations were Q58E (1.75%) and E57Q (1.23%), respectively (Table 3). Accordingly, the highest levels of resistance were observed against the drugs nevirapine (NVP), efavirenz (EFV), Lamivudine(3TC), and Emtricitabine (FTC) (S2-Fig in S1 Fig).

**Table 3:** Spectrum of major drug resistance mutations by antiretroviral drug class among patients with virologic failure in Chongqing, 2019–2023.

Drug Class	Mutation Type	Mutation	Number of Patients n(%)
**PIs(n = 1426)**		Any PIs	77(5.39)
	Major Mutations	M46I	22 (1.54)
		V82A	14 (0.98)
		I54V	17 (1.19)
		I84V	7 (0.49)
		I47V	3 (0.21)
		L90M	1 (0.07)
		G48A	1 (0.07)
		V32I	1 (0.07)
		I50V	1 (0.07)
		L76V	4 (0.28)
	Accessory Mutations	L10F	10 (0.70)
		L33F	5 (0.35)
		Q58E	25 (1.75)
		K43T	21 (1.5)
		K20T	2 (0.14)
		L23I	1 (0.07)
		L24I	1 (0.07)
**INSTIs(n = 653)**		Any INs	40(6.13)
	Major Mutations	E92Q	5 (0.77)
		Q148R	4 (0.61)
		N155H/S	4 (0.61)
		S230R	4 (0.61)
		G118R	3 (0.46)
		P145S	1 (0.15)
	Accessory Mutations	E157Q	8 (1.23)
		E138K/A	5 (0.77)
		L74M	4 (0.61)
		S147G	3 (0.46)
		G163R	3 (0.46)
		T97A	3 (0.46)
		G140K	1 (0.15)
		A128T	2 (0.31)
		H51Y	2 (0.31)
		Q95K	1 (0.15)
**NRTIs(n = 1426)**		Any NRTIs	636(44.60)
		M184V/I	542 (38.00)
		K65R/N	297 (20.83)
		K70E/T/N/R/Q	179 (12.55)
		D67N/E/G/T	137 (9.61)
		K219E/R/Q/N	91 (6.38)
		M41L	55 (3.86)
		L210W	74 (5.19)
		T215A/I/F/Y/	36 (2.52)
		Q151M	3 (0.21)
		F116Y	3 (0.21)
		F77L	1 (0.07)
		L74I/V	74 (5.19)
		S68N/G	52 (3.65)
		A62V	50 (3.51)
		V75I/M/A/T	38 (2.66)
		T69D/T	13 (0.91)
		E44D/A	10 (0.70)
		Y115F	137 (9.61)
**NNRTIs(n = 1426)**		Any NNRTIs	872(61.15)
		K103N/S/H	356 (24.96)
		V106M/I/A	267 (18.72)
		G190A/S/Q/E/T/V/C	176 (12.34)
		Y181C/V	157 (11.01)
		K101E/H/P	97 (6.80)
		Y188L/W/C/D/H	63 (4.42)
		L100I	53 (3.72)
		M230L	34 (2.38)
		A98G	24 (1.68)
		E138Q/A/G/K	68 (4.77)
		H221Y	35 (2.45)
		K238T/N	7 (0.49)
		V179D/E/L/T	326 (22.86)
		F227L/C	95 (6.66)
		P225H	72 (5.05)
		V108I	38 (2.66)
		L234I	11 (0.77)
		N348I	5 (0.35)
		K103R	2 (0.14)
		Y318F	11 (0.77)

note:a Mutations are categorized as Major or Accessory according to the Stanford University HIV Drug Resistance Database (HIVdb) and aligned with current expert consensus.b The total number of patients harboring any INSTI resistance mutation was 40. The mutation counts in this table represent the frequency of specific mutation sites; the sum exceeds the patient number because a single patient can harbor multiple mutations.c Similarly, 77 out of 1426 patients (5.4%) harbored at least one PI resistance mutation.d K103N, K103S, and K103H, all major NNRTI resistance mutations, are reported collectively (n = 356). The polymorphic variant K103R (n = 2) is listed separately under “Accessory Mutations”

INSTIs: Integrase strand transfer inhibitors; NNRTIs: Non-nucleoside reverse transcriptase inhibitors; NRTIs: Nucleoside reverse transcriptase inhibitors; PIs: Protease inhibitors

### Univariate versus multivariate analysis

Univariate analysis identified significant associations between the prevalence of DRMs and both baseline CD4 + T-cell count and the initial ART regimen (Table 4). The DRM rate was inversely associated with CD4 + T-cell count, being highest in patients with counts <100 cells/μL (68.67%, 561/817), intermediate in those with counts of 100−250 cells/μL (61.14%, 236/386), and lowest in those with counts ≥250 cells/μL (56.05%, 125/223; P < 0.05). Regarding the initial regimen, patients starting with an NNRTI-based regimen had the highest DRM prevalence (67.82%, 746/1100), significantly higher than those starting on PI-based (55.93%, 33/59), INSTI-based (53.33%, 32/60), or other/unknown regimens (all approximately 53−54%; P < 0.001). The specific drug combinations comprising these regimen categories are detailed in the footnote to Table 4. In contrast, no significant associations were found between DRM prevalence and sex, age, ART duration, or baseline HIV-1 RNA level (all P > 0.05).

**Table 4:** Univariate analysis of factors associated with drug resistance mutations among patients with virologic failure in Chongqing, 2019–2023.

Characteristics	Patients, n	DRMs prevalence, n (%)	*χ* ^*2*^	*P*
**Sex**				
Male	1093	707(64.68)	0.111	0.739
Female	333	215(64.56)		
**Age group (years)**				
<20	33	25(75.76)	6.121	0.295
20–29	157	97(61.78)		
30–39	297	190(63.97)		
40–49	264	178(67.42)		
50–59	315	211(66.98)		
≥60	360	220(61.11)		
**Baseline CD4** ^+^ **T-cell count(cells/μL)**				
<100	817	561(68.67)	12.112	**0.002**
100–250	386	236(61.14)		
≥250	223	125(56.05)		
**Baseline Log**_**10**_ **VL(copies/mL plasma)**				
<3	58	34(58.62)	1.752	0.625
3–4	231	155(67.10)		
4–5	412	271(65.78)		
≥5	725	462(63.72)		
**Initial ART regimen**				
2NRTIs+INSTIs	60	32(53.33)	28.612	**<0.001**
2NRTIs+NNRTIs	1100	746(67.82)		
2NRTIs + PIs	59	33(55.93)		
other	15	8(53.33)		
unknown	192	103(53.65)		
**ART duration (years)**				
<1	234	161(68.80)	3.340	0.188
1–5	831	538(64.74)		
≥5	361	223(61.77)		

Note:Detailed composition of the initial ART regimens: Among the 1,100 patients starting a 2NRTIs+NNRTIs regimen, the most common was TDF + 3TC + EFV, accounting for 65.0% (715/1,100). For the 60 patients on a 2NRTIs+INSTIs regimen, ABC + DTG + 3TC was the most frequent (15.0%, 9/60). Among the 59 patients on a 2NRTIs + PIs regimen, AZT + 3TC + LPV/r was predominant (15.3%, 9/59).The “other” category (n = 15) refers to a small number of patients who were initiated on non-standard regimens that did not conform to the conventional framework of two NRTIs plus a core agent (INSTI, NNRTI, or PI). Examples include regimens comprising a single NRTI combined with an NNRTI, or triple-NRTI combinations.The “unknown” category (n = 192, 13.5% of the cohort) indicates cases where the record of the initial ART regimen was missing from the medical charts during our retrospective data collection.

ART: Antiretroviral therapy, DRMs: Drug resistance mutations, HIV: Human deficiency virus, INSTIs: Integrase strand transfer inhibitors, NRTIs: Nucleoside reverse transcriptase inhibitors; NNRTIs: Non-nucleoside reverse transcriptase inhibitors, PIs: Protease inhibitors;TDF:tenofovir disoproxil fumarate,3TC:lamivudine,EFV:efavirenz,ABC:abacavir,DTG:dolutegravir,AZT:zidovudine,LPV/r:lopinavir/ritonavir (Table 4 continue)

Variables significant in univariate analysis were included in a multivariate logistic regression model, which confirmed baseline CD4 + T-cell count and initial ART regimen as independent predictors of DRMs. The model demonstrated good fit (Hosmer-Lemeshow test, P = 0.809) (S3-Fig in S1 Fig). Compared to patients with a CD4 + count <100 cells/μL, the odds of developing DRMs were significantly lower for those with counts of 100–250 cells/μL (aOR = 0.698, 95% CI: 0.541–0.901) and ≥250 cells/μL (aOR = 0.559, 95% CI: 0.411–0.760). Furthermore, patients initiating an NNRTI-based regimen had nearly twice the odds of developing DRMs compared to those starting an INSTI-based regimen (aOR = 1.933, 95% CI: 1.142–3.271) (Table 5).

**Table 5:** Multivariate logistic regression analysis of factors independently associated with drug resistance mutations among patients with virologic failure.

Variable	Estimate	Standard Error	Wald	P	OR (95% CI)
**Intercept**	0.288	0.264	1.197	0.274	
**Baseline CD4 + T-cell count (cells/μL)**					
<100					1.00(ref)
100–250	−0.360	0.130	7.632	**0.006**	0.698(0.541 ~ 0.901)
≥250	−0.582	0.157	13.762	**<0.001**	0.559(0.411 ~ 0.760)
**Initial ART regimen**					
2NRTIs+INSTIs					1.00(ref)
2NRTIs+NNRTIs	0.659	0.269	6.019	**0.014**	1.933(1.142 ~ 3.271)
2NRTIs + PIs	0.210	0.372	0.319	0.572	1.234(0.595 ~ 2.561)
other	0.083	0.583	0.020	0.887	1.086(0.347 ~ 3.405)
unknown	0.007	0.298	0.001	0.981	1.007(0.561 ~ 1.807)

CI, confidence interval; OR, odds ratio;INSTIs: Integrase strand transfer inhibitors; NNRTIs: Non-nucleoside reverse transcriptase inhibitors; NRTIs: Nucleoside reverse transcriptase inhibitors; PIs: Protease inhibitors

## Discussion

In this study, we observed a high overall burden of HIV-1 drug resistance, with DRMs present in 66.55% of the treatment-failure patients with successful pol gene amplification (n = 1,426); integrase gene data were available for a subset of 653 patients. This finding is consistent with a concerning national trend of rising drug resistance. For instance, a recent report from Henan Province documented an even higher resistance rate of 81.2% among patients with ART failure [15]. The rate in our cohort exceeds the 64.84% reported in Liaoning Province [16] and is substantially higher than the 39.83% documented in Hunan Province [17]. These marked regional variations within China highlight the uneven burden of drug resistance. When viewed in a global context, the drug resistance burden identified in our study aligns with findings from a regional cross-sectional survey in Uganda. Among patients with treatment failure, the HIV drug resistance rate reached 73.9%, and NNRTI resistance was the most prevalent form, affecting 66% of all failures [18]. This pattern highlights the inherently low genetic barrier of NNRTI-based regimens issue that persists across diverse geographic and socioeconomic settings.

The distribution of HIV-1 subtypes in our cohort, characterized by the predominance of CRF07_BC (50.14%), is consistent with our previous report [19]. The phylogenetic analysis confirmed this subtyping. The relatively low resistance rates observed for PIs (5.39%) and INSTIs (6.13%) in our study are primarily due to their high genetic barrier to resistance, coupled with their historically limited use in China. As of 2022, INSTIs were not widely covered by national free treatment programs or health insurance in many provinces, restricting their use and the concomitant clinical need for routine resistance testing [15]. This context was also relevant in Chongqing, where integrase inhibitor resistance testing was only prioritized and incorporated into routine practice after 2020.

Our findings reinforce the established link between advanced immunosuppression and the development of drug resistance. We confirmed that a lower baseline CD4 + T-cell count was an independent risk factor for DRMs [20,21]. Our multivariate analysis further substantiated the association between advanced immunosuppression and the development of drug resistance, confirming that a lower baseline CD4 + T-cell count was an independent risk factor for DRMs, consistent with findings from other studies [22]. This underscores the critical importance of early ART initiation and regular monitoring of VL and drug resistance (DR) in patients with low CD4 + counts to preempt treatment failure. Interestingly, and in agreement with some previous cohorts [15,20], we did not observe a significant association between high baseline HIV-1 RNA levels and the presence of DRMs. The analysis by drug class revealed a pronounced hierarchy in resistance prevalence, with NNRTI resistance being the most common (61.15%), followed by NRTI (44.60%), INSTI (6.13%), and PI (5.39%) resistance. Accordingly, the highest levels of predicted drug resistance were observed for the NNRTIs NVP (52.94%) and EFV (52.52%), and for the NRTIs 3TC and FTC (42.35%). This pattern is directly reflected in the initial ART regimen analysis, where patients starting with an NNRTI-based regimen had the highest incidence of DRMs (67.82%), significantly higher than those starting on INSTI-based (53.33%) or PI-based (55.93%) regimens. Logistic regression confirmed the initial regimen as an independent risk factor. The stark contrast between NNRTI-based and INSTI-based regimens can be attributed to the intrinsically low genetic barrier of NNRTIs, where a single mutation can confer high-level resistance, coupled with their historical predominance in first-line therapy [23–25]. In contrast, INSTIs possess a higher genetic barrier and are more forgiving of suboptimal adherence [26]. As INSTIs are increasingly adopted as first-line regimens globally and in China, our data provide a crucial baseline for future surveillance. Continuous monitoring of INSTI resistance is imperative to preserve the long-term efficacy of this drug class. Furthermore, within the INSTI class, the lower DRM incidence is likely driven by the higher resistance barrier and efficacy of second-generation inhibitors (DTG, BIC, CAB) compared to first-generation agents (EVG, RAL).

The profile of specific resistance mutations in our cohort reinforces these findings. The most frequent NRTI mutations were M184V/I (38.00%) and K65R/N (20.83%). M184V/I confer resistance to 3TC and FTC but do not affect tenofovir (TDF). However, the K65R mutation is associated with intermediate resistance to TDF. Therefore, while TDF-containing INSTI-based regimens remain a viable salvage strategy for patients without K65R, alternative NRTIs such as zidovudine (AZT) should be considered for those harboring this mutation. For NNRTIs, common mutations included K103N/H/S (24.96%) and V106M/I/A (18.72%). Specifically, K103N/H/S causes high-level resistance to efavirenz and nevirapine, necessitating a switch to INSTI- or PI-based regimens, which aligns with China’s treatment strategy to prioritize INSTIs where feasible. Strikingly, this mutation profile closely mirrors that reported in Uganda [27], highlighting the consistent selective pressure exerted by historically used NNRTI-based first-line regimens across diverse global settings. While this pattern is global, China’s expanding access to INSTIs offers a direct solution. It is also notable that a subset of patients experienced VF without detectable DRMs, strongly implicating suboptimal adherence as a major contributing factor.

Several limitations of this study warrant consideration. First, the retrospective design precluded the collection of detailed, quantifiable adherence data (e.g., from pharmacy refills or pill counts). As suboptimal adherence is a primary driver of virological failure (VF) and acquired drug resistance, its absence represents a key limitation and a potential unmeasured confounder in our risk factor analysis. Second, the monitoring frequency of HIV viral load was limited, as many patients primarily relied on the annually provided free national testing program. This infrequent monitoring may have delayed the detection of virological failure, potentially allowing for the accumulation of resistance mutations and affecting the accurate assessment of risk factors associated with the timing of failure. Third, the sample size for INSTI resistance testing was limited (n = 653), primarily because routine testing for INSTI resistance was only implemented after 2020 in our setting, and was further influenced by subsequent clinical and economic factors.

## Conclusions

Our study reveals a substantial burden of HIV-1 drug resistance among patients with treatment failure in Chongqing, driven primarily by low baseline CD4 + T-cell counts and the use of NNRTI-based initial regimens. To address this challenge, we recommend a strategic shift towards regimens with a higher genetic barrier, specifically advocating for DTG-based therapies as the preferred first-line option. In settings where DTG is temporarily unavailable, PI-based regimens represent a viable alternative, given the persistently low prevalence of PI resistance observed. Furthermore, to enable prompt detection of treatment failure, we strongly recommend increasing the frequency of routine VL monitoring to at least every 6 months for patients on stable ART, and more frequently (e.g., every 3 months) during the first year of treatment or following regimen switches. Regarding resistance testing, we emphasize the critical importance of performing systematic genotypic testing upon confirmation of virological failure (ideally within 4 weeks), rather than relying solely on baseline assessment. This timely intervention ensures guided regimen switching, balances clinical urgency with cost-effectiveness, and helps preserve future treatment options. These measures, coupled with enhanced adherence support, are essential to improve long-term treatment outcomes and curb the spread of drug-resistant HIV in the region.

## Supporting information

S1 Fig Phylogenetic tree of HIV-1 pol sequences. The tree was inferred using the neighbor-joining method in MEGA 11 under the general time reversible model with 250 bootstrap replicates. Different subtypes are shown in different colors. Reference sequences (GenBank No. U51189, AF286226, AF286229, AF069670, AY945737, DQ207940, U21135, AF067155, JX574661, AF077336, AF061642, AF190127, AF082395, AJ249235, AF286236) were downloaded from the Los Alamos HIV Sequence Database (https://www.hiv.lanl.gov/).
(ZIP)

## Abbreviations

3TC: Lamivudine
ABC: Abacavir
aOR: Adjusted Odds Ratio
ART: Antiretroviral Therapy
BIC: Bictegravir
CAB: Cabotegravir
CI: Confidence Interval
CRF: Circulating Recombinant Form
DR: Drug Resistance
DRMs: Drug Resistance Mutations
DTG: Dolutegravir
EFV: Efavirenz
EVG: Elvitegravir
FTC: Emtricitabine
HIV: Human Immunodeficiency Virus
HIV-1: Human Immunodeficiency Virus Type 1
HSX: Heterosexual Contact
IDU: Intravenous Drug Use
INSTIs: Integrase Strand Transfer Inhibitors
INT: Integrase
IQR: Interquartile Range
MSM: Men Who Have Sex with Men
MTCT: Mother-to-Child Transmission
NFATP: National Free Antiretroviral Treatment Program
NNRTIs: Non-Nucleoside Reverse Transcriptase Inhibitors
NRTIs: Nucleoside Reverse Transcriptase Inhibitors
NVP: Nevirapine
PIs: Protease Inhibitors
PLWHA: People Living with HIV/AIDS
PWID: People Who Inject Drugs
RAL: Raltegravir
VL: Viral Load
VF: Virologic Failure
WHO: World Health Organization
