# Supplementary figures and images for: HIV-1 drug resistance and associated risk factors in patients with antiretroviral therapy failure in Chongqing, China, 2019–2023
Source: PLoS One. 2026 Feb 24;21(2):e0342301. doi: 10.1371/journal.pone.0342301 (PMC12931755; doi:10.1371/journal.pone.0342301)

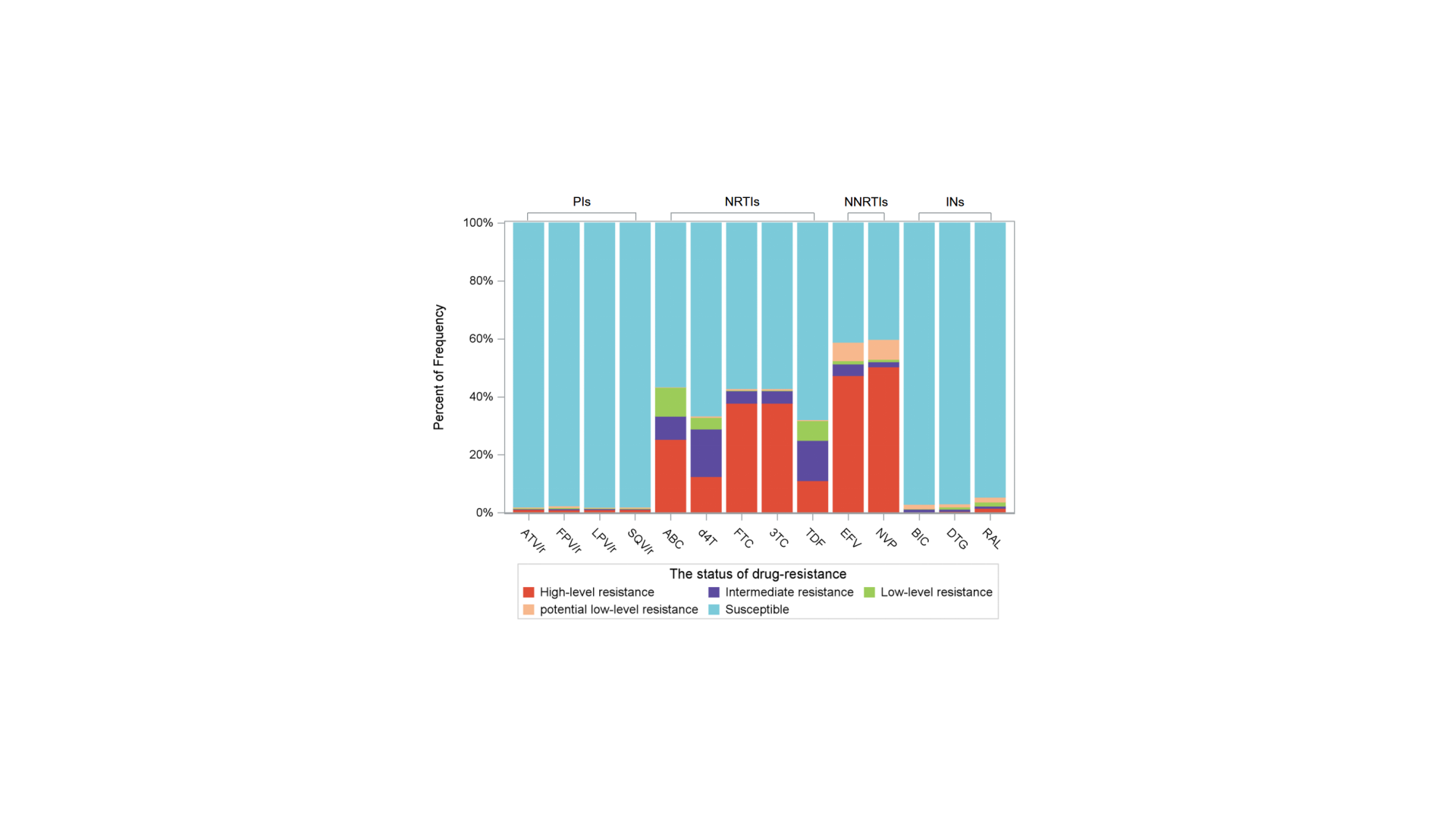

Supplement: S1 Fig — The tree was inferred using the neighbor-joining method in MEGA 11 under the general time reversible model with 250 bootstrap replicates. Different subtypes are shown in different colors. Reference sequences (GenBank No. U51189, AF286226, AF286229, AF069670, AY945737, DQ207940, U21135, AF067155, JX574661, AF077336, AF061642, AF190127, AF082395, AJ249235, AF286236) were downloaded from the Los Alamos HIV Sequence Database (https://www.hiv.lanl.gov/). (ZIP) [file pone.0342301.s001.zip › S2-Fig.tif]

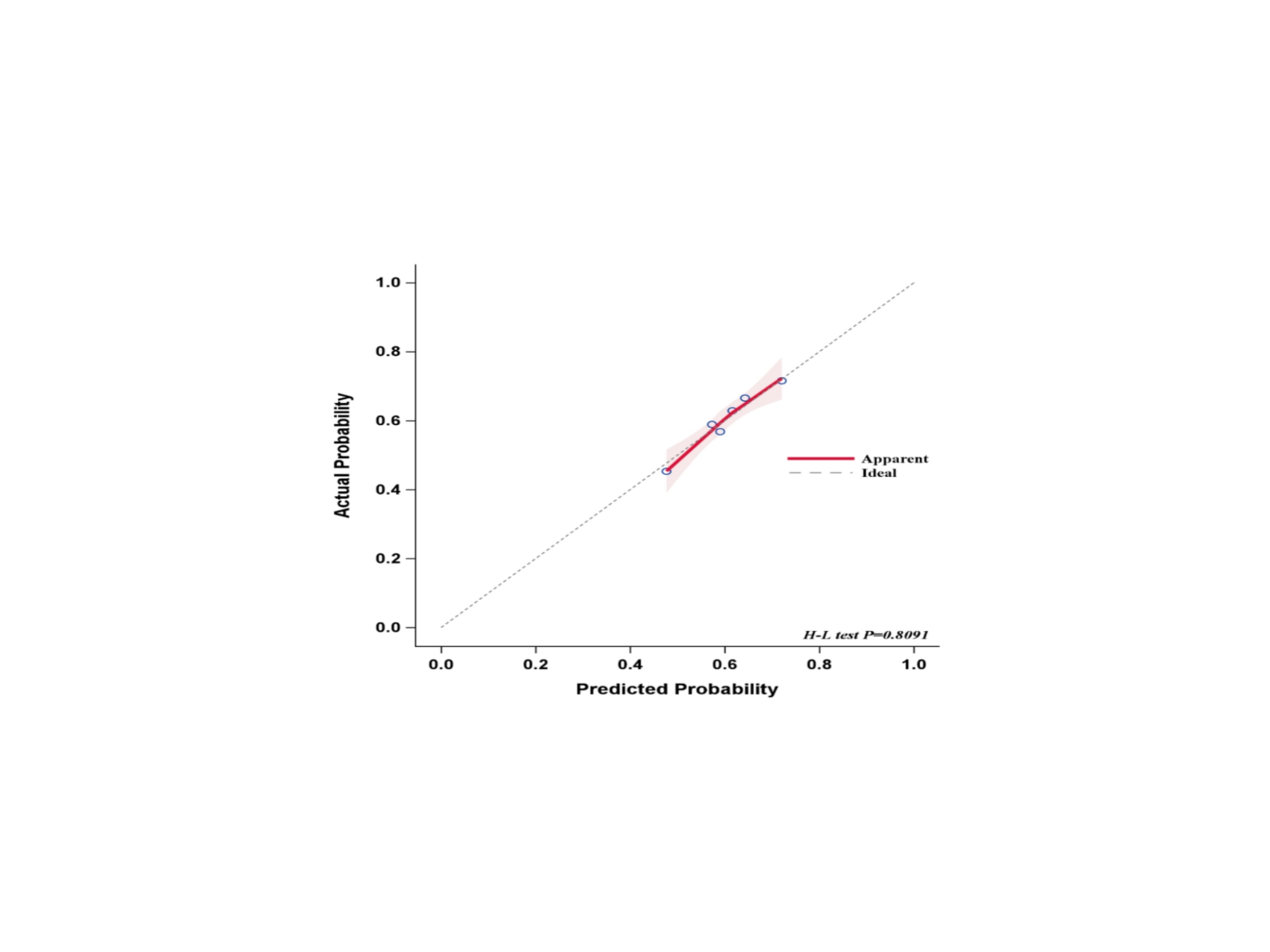

Supplement: S1 Fig — The tree was inferred using the neighbor-joining method in MEGA 11 under the general time reversible model with 250 bootstrap replicates. Different subtypes are shown in different colors. Reference sequences (GenBank No. U51189, AF286226, AF286229, AF069670, AY945737, DQ207940, U21135, AF067155, JX574661, AF077336, AF061642, AF190127, AF082395, AJ249235, AF286236) were downloaded from the Los Alamos HIV Sequence Database (https://www.hiv.lanl.gov/). (ZIP) [file pone.0342301.s001.zip › S3-Fig.tif]

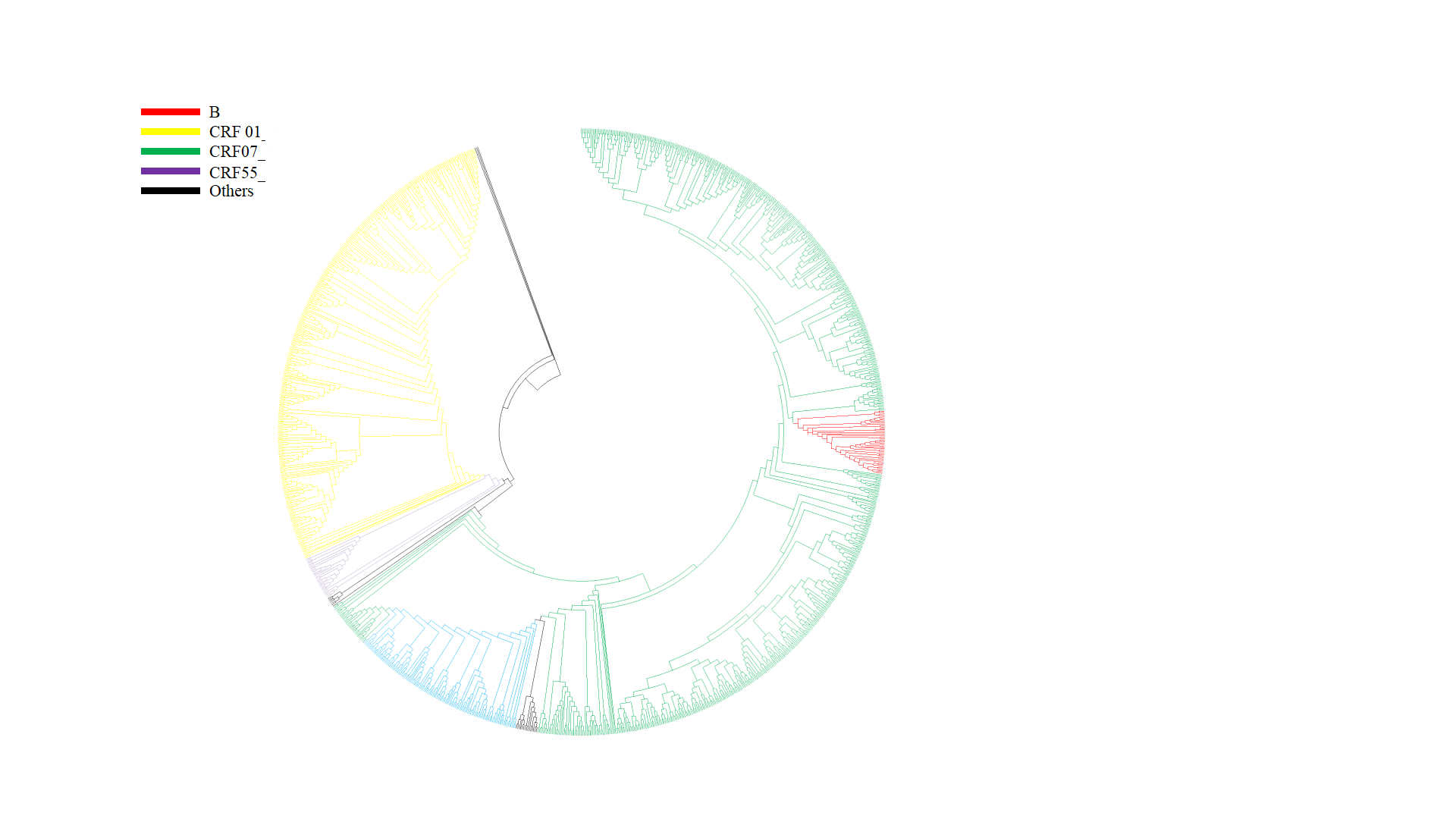

Supplement: S1 Fig — The tree was inferred using the neighbor-joining method in MEGA 11 under the general time reversible model with 250 bootstrap replicates. Different subtypes are shown in different colors. Reference sequences (GenBank No. U51189, AF286226, AF286229, AF069670, AY945737, DQ207940, U21135, AF067155, JX574661, AF077336, AF061642, AF190127, AF082395, AJ249235, AF286236) were downloaded from the Los Alamos HIV Sequence Database (https://www.hiv.lanl.gov/). (ZIP) [file pone.0342301.s001.zip › S1-Fig.tif]
